# A Manual of Otology

**Published:** 1898-11

**Authors:** 


A MANUAL OF OTOLOGY. By GORHAM BACON, A. M., M. D., Professor of Otology in Cornell University Medical College, New York. With an introductory chapter by CLARENCE J. BLAKE, M. D., Professor of Otology in the Harvard Medical School, Boston. Duodecimo, pp. 400, with 109 engravings and 1 colored plate. Philadelphia and New York: Lea Brothers & Co. 1898.

The student will welcome the appearance of Dr. Bacon's manual of otology, as the works on the diseases of the ear accessible to him, especially by American authors, are more extended than he needs. This manual contains the essential facts of otology as they are understood today, including the anatomy and physiology of the ear, its diseases and their treatment. The author is both an experienced teacher and practitioner and, with good discrimination, he has made a most judicious selection of the materials for the work. It is to be regretted, however, that the diction is not more lucid, and that the literary character does not correspond with the excellency of the material. It is to be hoped that in a second edition there will be less evidence of hasty production. A. A. H.

